# Regulation of Germ Cell mRNPs by eIF4E:4EIP Complexes: Multiple Mechanisms, One Goal

**DOI:** 10.3389/fcell.2020.00562

**Published:** 2020-07-07

**Authors:** Hayden P. Huggins, Brett D. Keiper

**Affiliations:** Department of Biochemistry and Molecular Biology, Brody School of Medicine, East Carolina University, Greenville, NC, United States

**Keywords:** eIF4E, 4EIPs, RBPs, translational control, germ granules, polyadenylation, deadenylation, mRNA decay

## Abstract

Translational regulation of mRNAs is critically important for proper gene expression in germ cells, gametes, and embryos. The ability of the nucleus to control gene expression in these systems may be limited due to spatial or temporal constraints, as well as the breadth of gene products they express to prepare for the rapid animal development that follows. During development germ granules are hubs of post-transcriptional regulation of mRNAs. They assemble and remodel messenger ribonucleoprotein (mRNP) complexes for translational repression or activation. Recently, mRNPs have been appreciated as discrete regulatory units, whose function is dictated by the many positive and negative acting factors within the complex. Repressed mRNPs must be activated for translation on ribosomes to introduce novel proteins into germ cells. The binding of eIF4E to interacting proteins (4EIPs) that sequester it represents a node that controls many aspects of mRNP fate including localization, stability, poly(A) elongation, deadenylation, and translational activation/repression. Furthermore, plants and animals have evolved to express multiple functionally distinct eIF4E and 4EIP variants within germ cells, giving rise to different modes of translational regulation.

## Introduction

Post-transcriptional regulation of mRNAs is frequently used to modulate gene expression throughout development in all plants and animals (Pushpa et al., 2017). Particularly during germ cell and embryo development, chromosome dynamics and/or spatial and temporal constraints may limit the ability of transcriptional regulation alone to adequately govern gene expression (Schier, 2007; Lebedeva et al., 2018). As a result, steady-state mRNAs levels often correlate poorly with their protein products in developing tissues (Becker et al., 2018). The question that arises is: How do germ cells selectively determine which mRNAs are repressed, degraded, or activated for translation in a spatial and temporal manner? Activation will ultimately produce the functional gene product and thus could be considered the more critical step in gene expression. Current models suggest that RNA-regulatory networks composed of RNA-binding proteins (RBPs), small non-coding RNAs, and translation initiation factors (eIFs) regulate nearly all aspects of mRNA metabolism (Nousch and Eckmann, 2013; Pushpa et al., 2017). Yet it remains unclear how these regulatory networks coordinate translational repression/activation switches of individual mRNAs in response to developmental stimuli. Failure to coordinate the translation of mRNAs encoding the many, sometimes opposing, signals results in aberrant development (Puoti et al., 2001). The beginning of an answer appears to be that mRNAs are assembled into specialized messenger ribonucleoprotein (mRNP) complexes as they transit from the nucleus. The assembled mRNP controls most aspects of the transcript’s life, including transport, localization, storage, translation, and stability. These protein-RNA complexes are hypothesized to be the discrete regulatory units of germline transcripts. Appropriate regulation is accomplished by remodeling mRNPs in germ granules or in the cytoplasm (see below). This broader model challenges earlier notions that focused on individual components of the complex, and their solitary roles in repression or decay. This review will provide a brief overview of some regulated mRNPs in various developmental models and draws parallels for their regulation between each organism. Although the individual mRNAs or their regulators may be different between systems, some common functional themes begin to emerge.

Translational repression of mRNAs by RBPs has been studied extensively; most bind to sequence specific elements within 5′ or 3′-UTRs to interfere with ribosome recruitment by blocking the translation initiation machinery or to recruit the Ccr4-Not1 deadenylase complex causing shortening of the poly(A) tail (Bhandari et al., 2014; Lahr et al., 2017; Hentze et al., 2018). The latter is generally accepted as the first step in targeted mRNA decay. Other RBPs, like PUF (Pumilio and FBF) family members, can also interact with components of the small-RNA pathway to interfere with the elongation step of protein synthesis by mechanisms that are not yet well understood (Friend et al., 2012). By contrast, a few RBPs act positively for expression. The Dazl group of proteins have been shown to stimulate the translation of their mRNA targets either directly or indirectly, by their interaction with poly(A)-binding protein (Collier et al., 2005). RBPs can therefore be thought of as modular regulators that recruit other factors to assemble more complex functional mRNPs. The composite mRNPs may have very different functions depending on cellular context, specific sequences within the mRNA, or association of other trans-acting factors.

The functions of small-RNA pathways on post-transcriptional control have also been well characterized, where they have been shown to mediate mRNA turnover, as well as inhibit translation through multiple mechanisms (Ding and Grosshans, 2009; Iwakawa and Tomari, 2015; Freimer et al., 2018). Broad regulation of post-transcriptional gene expression by small RNAs is beyond the scope of this review, but a comprehensive description of these mechanisms during germline development is found in Saxe and Lin (2011). Briefly, most miRNAs imperfectly match their targets and regulate gene expression post-transcriptionally by inhibiting translation or by initiating mRNA turnover via decapping and deadenylation (Carthew and Sontheimer, 2009). The mechanisms by which siRNAs induce gene silencing differ from those of miRNAs; siRNAs match their targets perfectly and use distinct Argonauts to induce endoribonucleolytic cleavage of their targets, i.e., “slicing” (Okamura et al., 2004). piRNAs interact with the PIWI class of Argonaut proteins and aid in distinguishing self from non-self (transposable element repression), transcriptional regulation, mRNA regulation, and transgenerational inheritance by similar “slicing” mechanism as those described for siRNAs (Weick and Miska, 2014). Interestingly, during *Drosophila* embryogenesis, transposon-derived piRNAs bind imperfectly to *nos* mRNA in an mRNP involving Aubergine, Smaug, and eIF4E complexes to initiate Ccr4-Not1 mediated deadenylation (Rouget et al., 2010) (see below).

**FIGURE 1:**
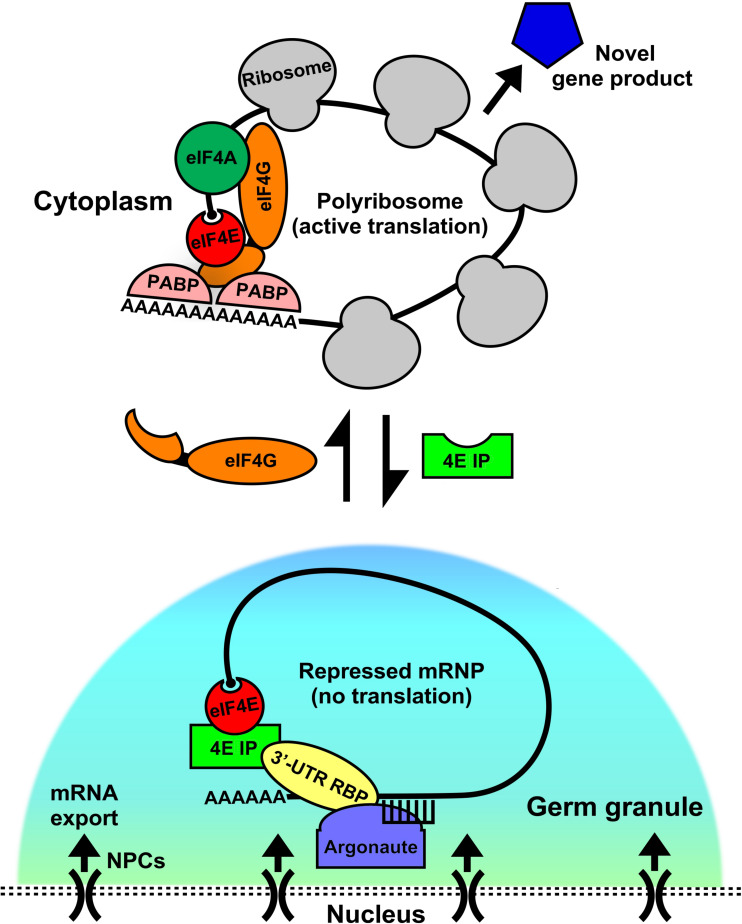
Germ granules as assembly sites for eIF4E:4EIP mRNP complexes to direct repression and activation of translation. In germ cells, mRNAs are exported out of the nucleus through the nuclear pore complex (NPC). As they exit they come under control by the germ granule environment. These conserved perinuclear structures act as hubs of post-transcriptional gene regulation, where they act as sites for the assembly and remodeling of diverse messenger ribonucleoprotein complexes (mRNPs). Germ granules are primarily sites of translational repression, as they do not contain ribosomes. However, recent evidence suggests that both repression and activation events may be set up here as a manner of mRNA sorting or licensing. Translation factors like eIF4E reside in complexes both within (repressed) and outside (activated) of the granule, potentially giving it a dual role. When eIF4E is in germ granules it binds to its cognate eIF4E-interacting protein (4EIP) in a complex with sequence specific 3′-UTR binding proteins. This complex prevents cap-dependent translation initiation by preventing eIF4G binding to eIF4E. Little is known about how the “decision” to activate translation is made, but our models suggests that a handoff from the granule to the cytoplasm occurs by mRNP remodeling. 4EIP is displaced by eIF4G and leads to initiation and recruitment to polyribosomes. Other factors participate in the repression to activation switch, like helicases (eIF4A, VASA, etc.) that unwind mRNA secondary structure, poly(A) polymerases (PAP) that extend 3′-poly(A) tails, and poly(A)-binding protein (PABP), which both protects transcripts and enhances translation via its interaction with eIF4G. Activating a repressed mRNP is the first step in producing novel gene products that can dictate the fate and function of germ cells.

Regardless of the repression mode, translational activation of the target mRNA must follow in order to introduce novel proteins and their functions, particularly in differentiating germ cells. For any developing cell type this is arguably the more critical step of post-transcriptional gene expression. Eukaryotic translation initiation factors (eIFs) play a fundamental role in the activation step via their ability to recruit the ribosome to repressed or stored mRNPs. Recent studies have begun to shed light on their unique roles in RNA-regulatory networks across diverse phyla. A model has emerged in which eIF4 factors reside in such mRNPs in both their repressed and active states (Figure 1). Data from metazoan and plant developmental systems are consistent with this model showing multiple eIF4 group members participate in mRNP remodeling and coordinate activation/repression switches that drive selective mRNA translation (Stebbins-Boaz et al., 1999; Yoffe et al., 2006; Mayberry et al., 2009; Hernandez et al., 2013; Mitchell and Parker, 2014; Morrison et al., 2014; Friday and Keiper, 2015; Friday et al., 2015; Lee et al., 2015; Huggins et al., 2020). This review focuses on known roles for isoforms of the mRNA cap-binding protein eIF4E, and cognate binding partners, the eIF4E-interacting proteins (4EIPs) in regulating mRNP localization, storage, turnover, and translational activation in germ cells and embryos (Figure 1). Recent studies show that germ cells often express multiple isoforms of eIF4E and 4EIPs (Hernandez et al., 2013; Huggins et al., 2020). Over the course of evolution, each has adopted specialized function in regulating translation during development. This review seeks to outline the contribution of eIF4E:4EIP complexes in germ cells and embryos that control translational repression/activation switches. Such switches are vital for the progression of germ cells throughout meiosis, for example (Friday et al., 2015; Lee et al., 2015). For clarity, the nomenclature 4E-interacting protein (4EIP) is used for all eIF4E-sequestering proteins other than the canonical 4EBPs 1 and 2 that are released upon phosphorylation by mTOR kinase. However, the 4EIPs may also regulate cap-dependent initiation, but in response to developmental rather than nutritional cues. A comprehensive understanding of how mRNP remodeling mechanisms are integrated within the cell to drive selective protein synthesis and cell fate decisions is critical to understanding the regulation of gene expression in germ cells.

## eIF4 and Regulation of Cap-Dependent Translation

Canonical members of the eIF4 complex are the first to bind mRNAs at the 5’ end and include: the m7Gppp cap-binding protein eIF4E; the scaffold protein eIF4G, which supports mRNA circularization via interactions with eIF4E and poly(A)-binding protein (PABP), while also bridging eIF4 to the ribosome and recruiting eIF4A, the DEAD-box RNA helicase used to unwind secondary structure (Gingras et al., 1999). Additionally, eIF4B stimulates eIF4A activity although it is not part of eIF4 *per se* (Sen et al., 2016). These factors assemble on mRNAs (that presumably are part of preformed mRNPs) and stimulate translational activation by recruiting the 43S activated small ribosomal subunit to form the 48S preinitiation complex (PIC). Subsequent 5′ to 3′ scanning of the untranslated region and recognition of the AUG start codon, followed by 60S subunit joining leads to productive protein synthesis. Initiation of most cellular mRNAs relies on recognition of the 5′ m7Gppp cap and therefore the eIF4E:eIF4G interaction. mRNAs that show strong cap-dependence (CD) are those with long and/or complex 5′-UTRs, and consequently rely on the complete, intact eIF4 complex for robust translation (Rozen et al., 1990). Because 48S PIC assembly is rate-limiting for translation, initiation rates of these structurally complex mRNAs benefit from eIF4A helicase activity as well as the circularization induced by eIF4E:eIF4G:PABP interactions between 5′ and 3′ ends of the message (Figure 1). It is now accepted that circularization leads to more efficient re-initiation and ribosome recycling following termination (Wells et al., 1998; Mancera-Martinez et al., 2017). Furthermore, eIF4E stimulates eIF4A helicase activity by binding an inhibitory region in eIF4G, indicating that eIF4E provides multiple activities within the eIF4 complex to stimulate translation of mRNAs with complex 5′ UTRs (Feoktistova et al., 2013). Other less complex mRNAs and those containing an internal ribosome entry site (IRES) translate cap-independently (CI) by binding eIF4G/eIF3 and the 43S PIC directly via conserved sequences or structural motifs (Komar and Hatzoglou, 2011). Internal binding mechanisms effectively circumvent the need for eIF4E-mediated recruitment; in many cases the eIF4E-binding region of eIF4G is cleaved off or missing in the CI mechanism. Importantly, the CI initiation mechanism is also utilized in animal germ cell development. However, it is beyond the scope of this review, but has been reviewed elsewhere (Keiper, 2019).

The balance between CD and CI synthesis at the eIF4E:eIF4G node is regulated by nutrients in somatic tissues by target of rapamycin (TOR) kinase via phosphorylation and inhibition of canonical eIF4E-binding proteins (4EBPs), which were among the first 4EIPs discovered but have a much narrower definition, and will not be the primary focus of this review (Beretta et al., 1996). Regulation of this node is consistent with the downstream effects of TOR activity, which simulates proliferation and differentiation via eIF4E-sensitive mRNAs like cyclins, VEGF, and Myc (Rosenwald et al., 1995; Kevil et al., 1996). TOR also contributes to global protein synthesis by phosphorylating p70 S6 kinase (S6K), which stimulates translation by phosphorylation and activation of RPS6 and eIF4B (Magnuson et al., 2012). Hypo-phosphorylated 4EBPs compete with eIF4G for binding the dorsal surface of eIF4E, through a structurally conserved alpha-helix motif (Tyr-X_4_-Leu-Φ: where X is variable and Φ is hydrophobic) (Beretta et al., 1996). Additional non-canonical eIF4E-binding motifs like those found in *Drosophila* 4EIPs (Thor, Cup, and 4ET) recognize the lateral surface of eIF4E, displacing eIF4G in preformed eIF4E:eIF4G complexes (Igreja et al., 2014). Thus, eIF4E-sequestering proteins are considered “broad-scale” translational repressors that disrupt eIF4E:eIF4G interactions/eIF4 formation and subsequent CD protein synthesis. However, translational control by TOR may also performs an mRNA-selective role in mammalian germ cells. Studies in mouse spermatogonia have shown that mTOR is required for differentiation and proliferation, presumably due to its activity in the retinoic acid (RA)-induced translational activation of mRNAs encoding KIT, SOHLH1, and SOHLH2. This provides an example of the translational machinery responding to a developmental stimulus (e.g., RA) by activating translation of distinct mRNAs (Busada et al., 2015). Whether or not translation of these mRNAs relies on mTORs ability to inhibit 4EBPs *per se* and promote CD synthesis remains unclear but seems likely. These regulatory paradigms involving eIF4E and 4EBP provide the cell with the ability to rapidly alter the ratio of CD to CI translation, and therefore selectively determine which pools of mRNA have a competitive advantage for being translated on ribosomes. The ability of a particular 4EIP to inhibit translation initiation via disruption of the eIF4E:eIF4G interaction should be addressed individually, but a number of 4EIPs, like *Drosophila* Cup, are able to compete with eIF4G for eIF4E binding and inhibit protein synthesis (see below). Whether or not certain 4EIPs are TOR regulated is largely unknown, yet remains an interesting question.

To add to the complexity to the broad CD vs. CI translational control outlined above, gene duplication events over evolutionary time have given rise to multiple isoforms of eIF4E and 4EIP in both plant and animal species (Jankowska-Anyszka et al., 1998; Rodriguez et al., 1998; Keiper et al., 2000; Hernandez et al., 2005). The evolution of reproductive schemes and embryonic development leading to diverse tissues in complex animals and plants appears to have taken advantage of these diversified isoforms for developmentally relevant translational control. eIF4E and 4EIP isoforms are often expressed in a tissue specific manner, and null mutation analysis in several species have shown that they have distinct non-redundant roles during development, particularly in germ cells (Rodriguez et al., 1998; Dinkova et al., 2005; Hernandez et al., 2005; Henderson et al., 2009; Song et al., 2010; Tettweiler et al., 2012; Huggins et al., 2020). Considering that these factors were classically thought to catalyze generalized mRNA initiation that allowed cells to maintain homeostatic levels of general protein synthesis, it is interesting that recent investigations show their unique developmental functions. Several interesting observations come out of the phenotypes derived from deficiencies in eIF4E and 4EIP genes in worms. One might expect that for proteins whose functions antagonize one another, opposite phenotypes should arise. Quite the contrary, the phenotypes caused by loss of each partner in the eIF4E:4EIP pair is remarkably similar, and each pair appears to facilitate a different developmental process (Kawasaki et al., 1998; Amiri et al., 2001; Sengupta et al., 2013; Huggins et al., 2020). In *C. elegans*, multiple eIF4E isoforms (IFE-1 – IFE-5) are expressed in a tissue specific manner and each has unique developmental functions (Keiper et al., 2000; Amiri et al., 2001; Dinkova et al., 2005; Song et al., 2010). The two major germ cell isoforms, IFE-1 and IFE-3, have distinct roles in translational regulation of unique subsets of mRNAs (Henderson et al., 2009; Friday et al., 2015; Huggins et al., 2020). By measuring translation of mRNAs in worms lacking either IFE-1 or IFE-3, it was found that IFE-1 is critical for translating specific mRNAs related to oocyte maturation (*pos*-1, *vab*-1, *mex*-1) and mRNAs involved in spermatocyte cytokinesis (Henderson et al., 2009; Friday et al., 2015). In fact, *ife*-1 mutant animals display defects in oocyte maturation and in sperm division, linking its biochemical function to phenotype (Henderson et al., 2009; Friday et al., 2015). IFE-3 on the other hand, is the canonical eIF4E-1 isoform from worms, yet is responsible for the translational repression of some germline sex-determination (GSD) mRNAs (*fog*-1, *fem*-3, *daz*-1), as observed by polysome profiling (Huggins et al., 2020). Importantly, this repression similarly relied on the IFE-3 cognate 4EIP, IFET-1 (4ET/Cup orthologue) suggesting that together they participate in a repressive mRNP that regulates this subset of mRNAs (Huggins et al., 2020). Importantly, both *ife*-3 and *ifet*-1 mutant worms show major defects in germ cell sex-determination, again linking their biochemical functions to their mutant phenotypes (Sengupta et al., 2013; Huggins et al., 2020). Interestingly, IFE-3 was found associated with the *C. elegans* Tudor domain protein TOFU-6 in 21U piRNA-generating mRNPs (Cordeiro Rodrigues et al., 2019). Whether or not IFE-3 represses GSD mRNA translation via piRNA biogenesis remains to be determined. Similarly, studies from *Drosophila* show that eight distinct eIF4E isoforms are expressed in diverse tissues (Hernandez et al., 2005). Each of these binds m7Gppp caps, however, eIF4E-6 and -8 bind with significantly lower affinity, due to conserved and non-conserved substitutions in the cap-binding region (Hernandez et al., 2005). Fly eIF4E-8 was found to be most similar to human and mouse eIF4E-2/4EHP type that was predicted to be unable to bind eIF4G and 4EIP (Hernandez et al., 2005). However, eIF4E-8 was shown to participate in a repression complex on Caudal mRNA by binding to the 5′ cap structure and the regulatory 3′-RBP Bicoid (Cho et al., 2005). This demonstrates an alternative eIF4E:4EIP mRNP repression mechanism that contributes to Caudal asymmetry in the egg. Expression of multiple eIF4E and 4EIP isoforms being co-opted during evolution to regulate translation of distinct mRNAs during germ cell development is not limited to nematodes and flies. Studies from vertebrate models, most notably mice and *Xenopus*, show that multiple eIF4E isoforms are expressed that exhibit similar use of 4EIPs to regulate the spatial and temporal translation or degradation of specific mRNAs via functional mRNP complexes (discussed below).

## Importance of Germ Granules in Translational Control

Germ granules are conserved large mRNPs that act as hubs of post-transcriptional regulation in the germline of most metazoans studied (Voronina et al., 2011). They represent an environment in which mRNAs exiting the nucleus come under stringent post-transcriptional regulation before entering the bulk cytoplasm. Additionally, mRNPs that form in the cytoplasm may transit to germ granules for remodeling and to adopt appropriate post-transcriptional control. Germ granules contain mRNAs that are important for development, small-RNAs that regulate message silencing, and RBPs that control storage, stability, and ultimately translation of resident transcripts (Sengupta and Boag, 2012). Importantly, germ granules in *C. elegans* appear distinct from P bodies, whereas in mouse spermatogonia evidence points to the structures being more closely related (Kotaja et al., 2006; Gallo et al., 2008; Schisa, 2019). Thus, the overlap in functions and associated factors one might expect when comparing P bodies to germ granules is species-dependent. It is generally recognized that P bodies and stress granules have major functions in mRNA turnover and translational stalling, respectively, while germ granules have a much broader role in regulating the exit of transcripts from the nucleus, interaction with small-RNA pathways, mRNP remodeling, and maintenance of germline integrity. Recently, an additional germ granule called a Z granule has been identified in *C. elegans* that is distinct from P granules and has unique functions in transgenerational epigenetic inheritance (Wan et al., 2018). It is likely that many or all of these granules have substantial crosstalk in the regulation and maintenance of mRNP metabolism in germ cells. The first insights into germ granule structure showed that where they are electron dense, non-membrane bound organelles that associate with the nuclear periphery and nuclear pore complexes (NPC) (Mahowald and Hennen, 1971; Sheth et al., 2010). Later advances in fluorescent microscopy led to a better understanding of the highly dynamic nature of these germ granules (Brangwynne et al., 2009). We now know that they are liquid-liquid phase separating condensates, which can exchange components rapidly with the cytoplasm (Wu et al., 2019). It is thought that germ granules act broadly to control formation, remodeling, and shuttling of smaller nuclear and cytoplasmic mRNPs so that they may come under the appropriate translational regulation (Figure 1). Conserved components of germ granules that regulate post-transcriptional regulation of mRNAs include: RNA helicases (Vasa and related DEAD box proteins), RISC (RNA-induced silencing complex) components like AGO1/CSR-1 (Argonaute), 3′-UTR RBPs that generally repress translation but may also activate translation (Nanos, Pumillo, and CPEB), eukaryotic initiation factors (eIF4E, eIF5B), and 4EIPs (4E-T, Cup, Maskin, PGL-1, Bicoid) (Gao and Arkov, 2013). This collection of factors uniquely equips germ granules with the ability to regulate the diverse fates of developmentally important mRNAs as they are shuttled into the cytoplasm. While most of the RBPs take part in repression or turnover, the eIFs are known to activate translation. Importantly, the absence of detectable rRNAs or ribosomal proteins suggests that they lack ribosomes and no active protein synthesis occurs within the granule (Schisa et al., 2001; Marnik et al., 2019). Therefore, a simple model for germ cell translational control is that the granule to cytoplasm exchange sets up translational repression/activation switches, where repression happens in the granule and mRNAs that are activated for translation leave the granule as activated mRNPs and join with ribosomes (Figure 1). Likewise, it is conceivable that the inverse transit occurs to sequester populations of mRNPs whose expression must be quenched for subsequent development (Figure 1). Such a model then begs the question of whether factors are used for both repression and activation, and how either function is selected. Furthermore, what mechanisms exist to ensure these functions occur in the correct spatial and temporal manner in the intricate steps of germ cell and embryo development. It is clear from recent studies, however, that eIF4Es and 4EIPs are integral to this process of activation and repression (Cho et al., 2005; Minshall et al., 2007; Ottone et al., 2012; Huggins et al., 2020).

Germ granules and RBPs have been extensively studied in the nematode, *C. elegans*. One constitutive component of the so-called “P granule” is a self-associating RGG-motif RBP named PGL-1 (Kawasaki et al., 1998). PGl-1 is thought to be involved in the repression of germ cell mRNAs. It enhances the translational silencing of mRNAs bound to the PUF protein FBF-2, indicating that repressive 3′-UTR-RBPs can use PGL-1 as a cofactor to repress translation (Voronina et al., 2012). PGl-1 is also the non-canonical cognate 4EIP of IFE-1 (Amiri et al., 2001). The sequestration of an eIF4E isoform indicates yet another way that RBPs in germ granules control the translation of distinct mRNAs, by inhibiting the CD translational machinery itself. PGL-1 maintains a close functional relationship with IFE-1; it dictates the localization of this eIF4E isoform to perinuclear granules in early germ cells, causes its migration during oocyte meiotic maturation, and releases IFE-1 from granules in late spermatocytes (Amiri et al., 2001; Huggins et al., 2020). We have suggested that PGL-1 helps IFE-1 to locate and bind its target mRNA, and simultaneously prevents IFE-1 from binding to eIF4G to initiate translation (Friday and Keiper, 2015), just as canonical 4EBPs are known to do in other systems (Stebbins-Boaz et al., 1999; Salaun et al., 2003; Wilhelm et al., 2003). It is clear that PGL-1 sequesters a substantial portion of IFE-1 from the bulk cytoplasm in early germ cells, and releases it later in gametogenesis. Although direct competition of IFG-1 (eIF4G) and PGL-1 for IFE-1 has not been demonstrated, PGL-1 is not found in polyribosomes, unlike IFE-1 and IFG-1 (Friday and Keiper, 2015). There is some suggestion that IFE-1 may also be involved regulation of spermatogenic mRNAs with Argonautes like CSR-1, ALG-3, and ALG-4 (Figure 1), though it is unclear if these are linked or sequential steps in mRNA licensing (Campbell and Updike, 2015; Andralojc et al., 2017; Reed et al., 2020).

## eIF4E:4EIP Complexes Distinctly Influence Differentiation

One of the clearest examples of the link between granule dynamics, translational control, and germ cell differentiation occurs in the latter stages of *C. elegans* germ cell development where multiple eIF4E:4EIP complexes control translation of distinct mRNAs and control cell fate. During the latter stages of spermatogenesis the 4EIP PGL-1 is rapidly degraded in secondary spermatocytes, releasing IFE-1 from the P granule into the bulk cytoplasm (Updike et al., 2014; Huggins et al., 2020). There IFE-1 (and its cargo mRNAs) have the opportunity to associate with ribosomes and activate translation (Henderson et al., 2009; Friday et al., 2015). Importantly, IFE-1 is the only eIF4E isoform in *C. elegans* that binds PGL-1, indicating that other eIF4Es may be subject to different modes of regulation or have alternative cognate 4EIPs (Amiri et al., 2001). Recent studies provide direct evidence of the latter. RBPs isolated from worm extracts in their native mRNP complexes show that another 4EIP, IFET-1, associates exclusively with IFE-3 *in vivo* (Spike et al., 2014). Furthermore, IFET-1 is responsible for proper IFE-3 localization to germ granules that are distinct from IFE-1:PGL-1 granules (Huggins et al., 2020). Importantly, these IFE-3:IFET-1 complexes control translation of germline sex-determination mRNAs which are not subject to control by IFE-1 (Friday and Keiper, 2015; Huggins et al., 2020). Similar examples of unique eIF4E:4EIP regulation are found in *Drosophila* germ cells, where the IFET-1 orthologue, Cup, facilitates eIF4E nuclear-cytoplasmic shuttling, localization and function in fly oocytes (Zappavigna et al., 2004). Cup is also required for posterior localization of several mRNAs (*osk*, *grk*, *nos*, *etc.*), and maintains their translational repression, which is critical for body axis patterning during embryogenesis (discussed below). It seems clear that all animal germ cells contain multiple unique eIF4E:4EIP complexes. Regulating the transit and activation of these complexes contributes to differential mRNA translational control used to drive physiological germ cell processes in divergent animal systems.

Both PGL-1 and IFE-1 are required for fertility at elevated temperatures, most critically affecting late sperm development. The interpretation of both the biological and biochemical data indicate that IFE-1 recruits a subset of mRNAs whose translation is necessary for completion of sperm (and oocyte) differentiation (Kawasaki et al., 2011; Friday and Keiper, 2015). Likewise, IFET-1 and IFE-3 are required for normal germline sex-determination, where they prevent ectopic translation of sperm fate mRNAs and enhance translation of at least one oocyte mRNA (Huggins et al., 2020). These represent just a few instances in which eIF4E:4EIP mRNPs participate in translational repression/activation switches and that controls germ cell and embryo development in quite divergent animal species.

## Linking Localization of mRNAs to Translational Regulation

**FIGURE 2:**
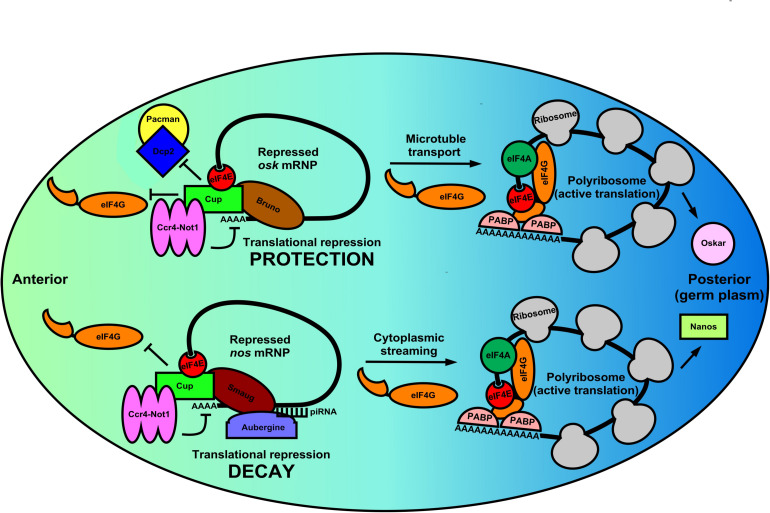
Germ cell mRNPs use the same eIF4E:4EIP core but different RBPs for altered modes of local translation in the *Drosophila* oocyte. During the latter stages of *Drosophila* oogenesis many germline mRNPs like *osk* and *nos* are localized to and specifically translated in the germ plasm (posterior pole). These regulated mRNPs use the eIF4E:4EIP core, but each employs a unique mechanism to control mRNA stability, localization, and eventual translation. In the anterior region of the oocyte *osk* mRNPs are circularized by a core complex containing the oocyte eIF4E, Cup (4EIP), and Bruno (3′-UTR-binding protein). Cup prevents eIF4E:eIF4G binding while simultaneously recruiting the Ccr4-Not1 deadenylase complex. Because eIF4E:Cup also prevents de-capping and 5′-3′ decay by the Dcp2/Pacman complex, *osk* mRNA is protected and translationally silent while being shuttled to the posterior pole via microtubules. Similarly, *nos* mRNPs in the anterior region of the oocyte are circularized by a eIF4E:Cup:Smaug complex, which again prevents translation initiation and recruits Ccr4-Not1. Unlike *osk*, however, *nos* mRNA localizes by cytoplasmic streaming that is inefficient. Therefore, Smaug recruits Aubergine and associated piRNA machinery to degrade *nos* mRNA outside the germ plasm. Little is known about the events that promote translational activation of these two distinct mRNPs. However, it is likely that a remodeling occurs in which Cup is displaced by eIF4G leading to polyribosome recruitment. Additionally, Oskar protein translationally activates the *nos* mRNP, indicating a complex interplay of translational regulation regimens that ensure that germ cell determinants are synthesized in correct time and place.

Localization of mRNAs is highly orchestrated in germ cells and embryos. A regimented set of sequence-specific RBPs bound to mRNAs to be localized often coincides with spatial and temporal translational regulation of the transcript (Suter, 2018). These events also rely on eIF4E:4EIP complexes for proper localization and correct message utilization (Romasko et al., 2013; Davidson et al., 2016). The clearest examples are found during *Drosophila* oocyte development and early embryogenesis. *oskar* mRNA (*osk*) becomes enriched in the posterior pole (germ plasm) during mid-oogenesis and *nanos* mRNA (*nos*) becomes enriched in the same region during late oogenesis (Figure 2). Other fly mRNAs such as *gurken* (*grk*) and *bicoid* (*bcd*) also exhibit unique and dynamic localization during the maternal-zygotic transition (MacDougall et al., 2003; Lasko, 2012). Each mRNA is simultaneously subject to complex translational control that restricts their regional expression. The protein products form a morphogen gradient in the oocyte or embryo that is critical for anterior-posterior differentiation. However, the process by which each is localized and translationally regulated appear to differ in each case, suggesting that multiple mechanisms are used during development to control spatial and temporal abundance of mRNAs and their translation. Such processes are not restricted to invertebrates. Similar examples can be found in vertebrate models, such as in *Xenopus* oocytes, where Vg1, VegT, Nanos-1 mRNAs all localize to the vegetal pole of the frog oocyte (Nijjar and Woodland, 2013).

The combination of mRNA localization and translational control by these mRNP complexes is of critical developmental importance. For example, mis-localization of regional *osk* mRNA translation during oogenesis results in embryos that lack an abdomen and have no germline (Kim-Ha et al., 1991). *osk* is also subject to complex translational control mediated by the oocyte eIF4E:Cup complex to prevent protein accumulation in the anterior portion of the oocyte. Suppression of initiation events is maintained by multiple mechanisms as the mRNP is being properly localized to the germ plasm (Nakamura et al., 2004; Igreja and Izaurralde, 2011). Cup is recruited to *osk* mRNA by Bruno, which both represses and protects the transcript from degradation (Figure 2). Interestingly, the complex recruits the Ccr4-Not1 deadenylase complex to shorten the poly(A)-tail but does not destabilize the mRNA (Igreja and Izaurralde, 2011). Interestingly, translational repression by Cup does not appear to require the canonical eIF4E-binding motif, but instead translational repression and transcript protection rely on secondary non-canonical eIF4E binding motifs (Igreja and Izaurralde, 2011). Evidence shows that the eIF4E:Cup interaction prevents degradation of *osk* by directly blocking de-capping of the mRNA (Igreja and Izaurralde, 2011). Similar to Cup, recent evidence in human cells shows that eIF4E-T (4E-T) is able to repress translation and recruit Ccr4-Not1 deadenylase complex while preventing de-capping and targeted decay, preserving the mRNA in a repressed form (Raesch et al., 2020). Therefore, the eIF4E:4EIP cap-binding complex is necessary to protect the mRNA while it is coming under appropriate repression by the Ccr4-Not1 machinery in multiple systems. Once *osk* mRNP is properly localized to the posterior region of the oocyte, pre-bound eIF4E in the mRNP joins with eIF4G to initiate translation. Regional synthesis of functional Oskar protein directs patterning in the early embryo. Regulation of *osk* mRNA localization and translation are therefore a stepwise process involving repression/protection, transport, and ultimately translational activation in its complete program. All steps use the eIF4E:4EIP complex to ensure proper timing of the repression/activation switch. Complexes that likely first assemble in the nucleus co-transcriptionally or during splicing also play a role in setting up the localization/fate of mRNPs as they enter the cytoplasm and germ granules. Tsunagi and Magonashi are core components of the exon-junction complex (EJC) that marks mature mRNAs at those ligation points as they exit the nucleus (Parma et al., 2007). Both proteins are required for *osk* mRNA microtubule-dependent localization, indicating that 3′-UTR sequences do not act alone to localize mRNAs during development (Mohr et al., 2001).

In contrast to the efficient localization seen with *osk* mRNA, *nos* mRNA localization appears to be inefficient. Only a 4% enrichment of the mRNA in the posterior end of the early embryo is observed (Bergsten and Gavis, 1999). Consequently, restriction of Nanos to the germ plasm is accomplished by different means, involving both translational repression and targeted mRNA decay (Rouget et al., 2010; Lasko, 2012). Specifically, Smaug binds a 90-nt element within the 3′-UTR of *nos* mRNA deemed the translational control element (TCE) (Crucs et al., 2000). In the circularized mRNP structure, Smaug interacts with the Cup:eIF4E (Figure 2). This interaction is required for Smaug-dependent translational repression of *nos* mRNA indicating repression at the level of initiation, as well as regulation by Ccr4-Not1 (Nelson et al., 2004). Thus, *nos* mRNA appears to be repressed and localized by inhibition of CD initiation and targeted mRNA decay outside of the germ plasm, quite different from the repression, protection, and active transport regulation seen with *osk* mRNA, yet still utilizing eIF4E:4EIP complexes. Once synthesized in the germ plasm, Oskar protein takes part in the translational activation of repressed *nos* mRNPs by inhibiting deadenylation, indicating there is a complex interplay of germline determinants within the mRNP translational control network in fly oocytes (Zaessinger et al., 2006).

Additional insights into these translational repression/activation switches that rely on localization can be found in the regulation of Gurken mRNA (*grk*). During egg development *grk* mRNA also relies on Cup for proper localization, but in contrast to *osk* and *nos* mRNAs, it localizes to the dorsal anterior portion of the egg (Clouse et al., 2008). Developmental defects and biochemical analysis show that a closed-loop mRNP complex involving eIF4E, Cup, Bruno, and Squid mediates translational repression of *grk* mRNA that has not localized anteriorly. This repressive eIF4E:4EIP complex becomes activated for translation initiation by PABP55B-mediated recruitment of eIF4G once properly localized to the dorsal anterior region (Clouse et al., 2008). Mechanisms of *osk* and *grk* mRNA translational repression and localization have mechanistic parallels, even using many of the same *trans*-acting factors. So how is it possible that these shared factors are distinguised in order to localize these two transcripts to different regions of the egg? A protein named Encore, which binds to PABP55B may be key in answering this question (Clouse et al., 2008). Importantly, Encore is involved in the proper localization and translational activation of *grk* mRNA, but has no role in *osk* mRNA localization and activation. This suggests that each mRNP has *trans*-acting factors that provide context for activation. What is clear is that once mRNPs are properly localized, initiation factors like eIF4E and eIF4G are responsible for ribosome recruitment. However, the steps necessary and sufficient for initiation factors to overcome repressive trans-acting factors are largely unknown.

Similar examples of mRNP network crosstalk that leads to cell fate decisions can be found in *C. elegans* germline sex-determination (Puoti et al., 2001). Here too, mRNA localization and translational control pathways work in conjunction with one another to ensure proper *de novo* synthesis of specific gene products regionally. Importantly, even after each mRNA is localized to the correct place at the correct time, the necessary synthesis of the protein products has still not been accomplished. We know very little about the ensuing translational activation of repressed, localized mRNPs (Figure 2). What is clear is that members of the translation initiation apparatus already reside in the mRNP complex during repression and transport. Germline eIF4E isoforms appear to be ubiquitous in these repressed mRNPs and warrant further investigation as they are likely to be a driving force behind activation.

## Polyadenylation/Deadenylation Also Modulate Germ Cell Translational Control

**FIGURE 3:**
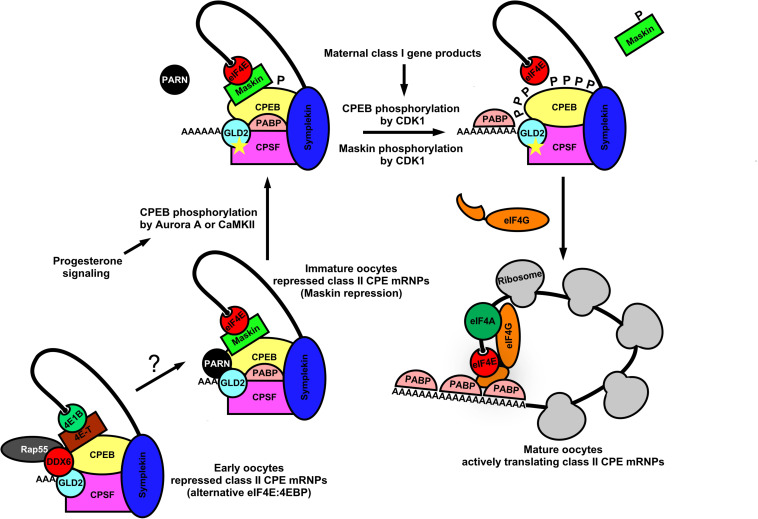
Dynamic mRNP remodeling promotes alternate mRNA repression, poly(A) elongation, and ribosome recruitment during *Xenopus* oocyte maturation. In immature *Xenopus* oocytes, class II CPE mRNAs are translationally repressed by eIF4E:Maskin cap-binding complexes directed by the CPEB, CPSF, Symplekin 3′-UTR-binding module. These complexes also contain PABP, PARN, and GLD2 indicating the potential for both poly(A) elongation and deadenylation. At the 5′-end, Maskin (4EIP) inhibits cap-dependent initiation by competing with eIF4G for eIF4E binding. At the 3′-end PARN shortens the poly(A) tail. Although GLD2 is resident within the complex, PARN activity appears to be dominant. It also may be that CPE-containing mRNAs cycle between deadenylation and polyadenylation. Upon progesterone stimulation, CPEB is phosphorylated by Aurora A kinase or CaMKII which leads to remodeling of the complex and ejection of PARN. GLD2 then catalyzes extension of the mRNA poly(A) tail. Concurrently, class I CPE containing mRNAs are translated and lead to activation of CDK1. CPEB and Maskin are phosphorylated by CDK1, and PABP is recruited to the poly(A) tail for further stabilization. Maskin is displaced and PABP associates with eIF4G, allowing more opportunity for a productive eIF4E:eIF4G interaction. This stepwise remodeling leads to translational activation of mRNAs critical for germinal vesicle breakdown and maturation. In early *Xenopus* oocytes CPE containing mRNAs are repressed by an alternative eIF4EB1:4E-T cap-binding complex along with P body components (DDX6 and Rap55), CPEB, CPSF, and scaffold protein Symplekin. PARN is not found in this complex, suggesting that translational repression is caused by inhibition of cap-dependent initiation. P body components resident may also act to degrade CPE these early class II transcripts.

In germ cells, deadenylation occurs mostly via the Ccr4-Not1 complex (Temme et al., 2004; Nousch et al., 2013; Waghray et al., 2015). The activity of this multi-subunit complex represents a major mechanism by which germ cells regulate the stability, localization, and translatability of mRNAs. Poly(A) removal may also be mediated by a family of proteins called PARNs, which require removal of the 5′-cap structure (discussed below). The examples cited above from *Drosophila*, involving *osk* and *nos* mRNAs, shows that mechanisms of poly(A)-regulation are used in oocytes and embryos to control the local translation of messages that are required to establish the germ plasm. Similar examples can be found in nematodes, frogs and mice, indicating the prevalence of poly(A)-mediated mRNA regulation throughout germ cell biology (Berthet et al., 2004; Temme et al., 2004; Nousch et al., 2013; Waghray et al., 2015). Transcripts may also have existing poly(A)-tails elongated in the cytoplasm in order to activate translation initiation and/or enhance transcript stability (Richter, 1999; Mendez and Richter, 2001). The choice to remove poly(A) tails or elongate them on an mRNA in a sequence-specific manner imposes another translational switch that may behave similar to a rheostat, controlling the overall “current” of translation (enhancing initiation frequency leading to greater total protein output on those mRNAs with elongated poly(A)-tails). It is now clear that eIF4E:4EIP complexes actively participate in poly(A) regulation as well (Cao and Richter, 2002; Wong and Schedl, 2011; Sengupta et al., 2013; Waghray et al., 2015). Polyadenylation of nascent mRNA transcripts first occurs co-transcriptionally in the nucleus. Cleavage of the nascent-mRNA downstream of the polyadenylation signal (PAS) by cleavage and polyadenylation specificity factor (CPSF) precedes addition of 200–250 adenine residues by nuclear poly(A)-polymerases (PAPs) (Wahle, 1995; Vi et al., 2013; Casanal et al., 2017). Poly(A)-tail formation is coordinated with transcription termination and nuclear export (Huang and Carmichael, 1996). Polyadenylation also takes place in the cytoplasm of germ cells and embryos and is highly regulated in order to control the balance of translational activation vs. mRNA decay (Sheets et al., 1995; Kim et al., 2010; Dufourt et al., 2017). Poly(A)-extension in the cytoplasm of germ cells is catalyzed predominantly by a highly conserved cytoplasmic poly(A)-polymerase, GLD-2 (Kim et al., 2010; Cui et al., 2013). Enhancing poly(A)-tail length promotes binding and multimerization of poly(A)-binding protein (PABP), which in turn facilitates mRNA closed-loop formation and increased translation through a direct interaction with eIF4G in the eIF4E:eIF4G cap-binding complex (Wakiyama et al., 2000; Kahvejian et al., 2001). Multimerization of PABP along the poly(A)-tail also serves to protect transcripts from targeted decay (Wormington, 1993; Vazquez-Pianzola et al., 2011). This dynamic may be more complicated, however, as recent evidence shows an unexpected dual role for PABPs in mediating both protection and poly(A)-decay, depending on which accessory factor it recruits, deadenylases and TOB proteins (Yi et al., 2018). Dynamic changes in poly(A)-tail length and subsequent PABP multimerization can remodel mRNPs quite dramatically and lead to nearly a 100-fold increase in translation initiation rate in response to developmental stimuli (Wakiyama et al., 2000; Kahvejian et al., 2001). This process is most well understood for mRNAs containing U-rich cytoplasmic polyadenylation elements (CPEs) in *Xenopus* oocytes (Figure 3). There are at least four subclasses of CPEs active during the oocyte to embryo transition in the frog; these are classified according to the developmental step at which they are used (Simon et al., 1992; Paillard et al., 2000). Not all poly(A)-regulated maternal mRNAs are polyadenylated simultaneously in the maturing oocyte. Therefore the selective activation of groups of CPE-containing mRNAs at various times during development ensures the proper progression of meiosis and sets up the egg to embryo transition (Pique et al., 2008). The most recognized CPE-containing mRNAs encode cell cycle regulators (Cyclins A, B, E, and CDK2) and the proto-oncogene c-Mos (Sheets et al., 1995; Stebbins-Boaz et al., 1996; Barkoff et al., 1998, 2000; Slevin et al., 2007). The sequence of events worked out by several labs is as follows: Cytoplasmic polyadenylation element binding protein (CPEB) binds the CPE of target mRNAs, a step essential for progesterone-induced oocyte meiotic maturation (Hake and Richter, 1994). During *Xenopus* oocyte maturation, CPEB acts as a central factor in large closed-loop mRNPs containing CPSF, Symplekin, eIF4E, and a 4EIP called Maskin to mediate translational repression of CPE containing mRNAs (Figure 3) (Stebbins-Boaz et al., 1999; Barnard et al., 2004). Maskin binds eIF4E to compete with eIF4G and inhibits translation at the step of ribosome recruitment (Stebbins-Boaz et al., 1999; Cao and Richter, 2002). Somewhat surprisingly, these large mRNPs also contain a PARN isoform and GLD-2, indicating the potential to carry out both deadenylation and poly(A)-elongation respectively (Kim and Richter, 2006). It may be that CPE-containing mRNAs cycle between deadenylation and polyadenylation states due to the opposing action of PARN and GLD-2, or that either may be exclusive on a single substrate mRNA, or even that PARN inhibits GLD-2 activity enzymatically or sterically. Thus, translational repression in quiescent *Xenopus* oocytes operates at both ends of the mRNA; inhibiting access to eIF4E on the cap and preventing enzymatic activity on the 3′-poly(A)-tail. With the induction of meiotic maturation, CPEB becomes phosphorylated on multiple residues, in response to progesterone signaling (Mendez et al., 2000). Prevailing models suggest that a cascade of mRNP remodeling events culminates in ejection of PARN from the complex, freeing the GLD-2 polymerase to execute poly(A)-elongation (Figure 3). The extended tail recruits more PABP, which in turn binds and recruits eIF4G. Tethered eIF4G provides more opportunity for a productive eIF4E:eIF4G interaction by enhancing proximity, and allowing it to displace Maskin. In younger oocytes PARN and Maskin are not detectable, so other mechanisms are in place to set up very early CPE-dependent repression of maternal mRNAs (Copeland and Wormington, 2001; Minshall et al., 2007). Interestingly, a biochemically separable repression complex was identified in immature oocytes that includes the eIF4E1b isoform (oocyte/embryo specific), eIF4E-T (4EIP, 4E-T), DDX6, and Rap55 (Figure 3). eIF4E1b binds only weakly to m7G-caps and does not appear to interact with eIF4G or Maskin (Standart and Minshall, 2008). Thus, in the absence of PARN, repression appears to be mediated by a eIF4E1b:eIF4E-T complex that prevents recruitment to ribosomes, similar to *nos* mRNA repression in *Drosophila* oocytes and in humans as outlined above (Figure 2). Indeed, several studies in other model organisms show that eIF4E isoforms can act as translational repressors. The eIF4E homologous protein (4EHP) takes part in Bicoid-dependent repression of *cad* mRNA during fly embryogenesis (Cho et al., 2005), and IFE-3 (the eIF4E1 ortholog) mediates IFET-1 (eIF4E-T)-dependent translational repression of germ line sex-determination mRNAs (*fog*-1 and *fem*-3) in worms (Huggins et al., 2020). Additionally, eIF4E:4EIP complexes have been linked to translational repression and mRNA decay during the maternal-zygotic transition in mice, where an oocyte-specific eIF4E was shown to interact with the 4EIP, BTG4, and recruits a catalytic subunit of the Ccr4-Not1 deadenylase complex (Yu et al., 2016).

Clearly, the mechanisms that control translational repression/activation switches in maturing *Xenopus* oocytes are complex, but like other animal germ cells they utilize eIF4E:4EIP complexes to mediate translation repression/activation switches. Simple thematic paradigms can be constructed from the modes of action that are conserved across most species. Early germ cell translational repression modes rely on preventing eIF4E:eIF4G interactions and thereby inhibiting cap-dependent translation initiation. Late germ cell repression modes not only inhibit the cap-dependent mechanism, but also remove poly(A)-tails, which reduces translational efficiency or enhances degradation. Thus, germ cell systems institute translational repression early, then maintain that repression until intrinsic or extrinsic signals drastically change cell fate (e.g., maturation). The signaling cascades activate translation by stimulating the eIF4 factors already resident in the mRNP and thereby disrupt the repressive effects of 4EIPs and RBPs, and activate GLD-2 to elongate the poly(A)-tails of their bound mRNAs.

## Conclusion and Future Perspectives

Germ cells and embryos must modulate gene expression to promote proper progression through development and ensure the survival of critical lineages of cells. While transcriptional regulation is generally the most efficient way to control gene expression in somatic cells, there is a necessity in germ cells to control when and where mRNAs are repressed, degraded, or activated for translation in order to complete their developmental fates. From germ cells we have learned that the functional units of translational control appear to be multicomponent mRNPs, which often include RBPs, small RNAs, eIF4E isoforms, and 4EIPs. Present in most animals studied, germ granules serve as conserved hubs of mRNA metabolism and contain many diverse mRNPs that function over the mRNA lifetime. There is strong evidence that germ granules contain (or maintain) mRNA-repressive structures because many of their components have prescribed roles in translational repression or mRNA decay (Sengupta and Boag, 2012). However, there is emerging evidence that both repression and activation may be pre-programmed within the germ granule, since they often contain not only repressive RBPs and small-RNA machinery, but also positive acting factors that contribute to mRNA stabilization and ribosome recruitment (Amiri et al., 2001; Thomson et al., 2008; Huggins et al., 2020). Indeed, germ granules must localize, protect, and store transcripts important for maintaining germline integrity (Schisa et al., 2001; Flemr et al., 2010; Voronina et al., 2011; Ouyang et al., 2019). What remains poorly understood is: How does the selection of either repression or activation on an mRNA or group of mRNAs begin in the granule? The question is critical in all dynamic developmental systems (e.g., learning neurons, stem cells, germ cells, embryos) responding to intrinsic and extrinsic signals by turning expression “on” and “off” by means of post-transcriptional regulation. Much has been learned about individual mRNP factors that control individual repression/activation switches, with a large emphasis on repression by RBPs and small-RNAs. This is perhaps most evident in seminal work on PUF-protein binding landscapes within the linear germline of *C. elegans*. Here stem cell proliferation, meiotic entry, and differentiation are all controlled by a remarkably small number of regionally expressed RBPs that bind 3′-UTRs of a subset of mRNAs to repress their translation (Prasad et al., 2016). Yet this does not fully explain how mRNAs are turned “on” when they progress beyond the repressive regions. Recently a greater appreciation has been gained for the other half of such regulatory switches, activation. Activating an mRNP for translation is arguably the more important step for introducing new activities into germ cells. At the heart of activation is the rate-limiting step of translation initiation catalyzed by eIF4E, eIF4G, and eIF4A, which recruit mRNAs to the ribosome (Hershey et al., 2012).

The regulated activation of mRNPs by the translational initiation machinery has been especially understudied in germ cells. Remarkably, studies from nematodes, flies, and frogs indicates that the eIF4 group factors are playing very specific developmental roles. Interestingly, a theme emerges from all animals and plants investigated, that multiple isoforms of each factor are expressed during development, providing the opportunity for each isoform to evolve a specialized function over time (Jankowska-Anyszka et al., 1998; Rodriguez et al., 1998; Keiper et al., 2000; Hernandez et al., 2005; Evsikov et al., 2006). Indeed, many have gained novel functions, as studies now show definitively that different isoforms of eIF4E and eIF4G have non-redundant developmental and biochemical roles and regulate distinct mRNA populations (Rodriguez et al., 1998; Keiper et al., 2000; Dinkova et al., 2005; Baker and Fuller, 2007; Franklin-Dumont et al., 2007; Contreras et al., 2008; Henderson et al., 2009; Song et al., 2010; Hu et al., 2018; Keiper, 2019; Huggins et al., 2020). Among these, the appearance of germ-cell specific mRNA cap-binding protein eIF4Es is a common theme. In this review we have put special emphasis on germ cell eIF4Es and non-canonical forms of its regulatory interacting proteins (4EIPs). Interestingly, eIF4E was initially characterized biochemically as an exclusively positive acting catalytic translation initiation factor (Rhoads et al., 1993; Sonenberg and Gingras, 1998). The first identification of canonical 4EIPs showed that they sequestered eIF4E from eIF4G, thereby blocking initiating activity (Haghighat et al., 1995; Mader et al., 1995). Recent investigations have led to the discovery of many classes of 4EIPs that form large mRNPs (Nelson et al., 2004; Zappavigna et al., 2004; Hernandez et al., 2013; Sengupta et al., 2013; Igreja et al., 2014). Because eIF4E is found to reside in repressive (with 4EIPs) and active (with eIF4G) mRNP complexes, it strongly suggests that the mRNA cap-binding protein indeed has roles as both an activator and repressor of translation depending on cellular and sequence-specific contexts. Indeed, we recently showed that the loss of one eIF4E isoform (IFE-3) in *C. elegans* caused very specific translational de-repression of certain mRNAs involved in germline sex-determination (Huggins et al., 2020). Likewise, the eIF4E2 (4EHP) isoform was shown to interact with Bicoid to facilitate translational repression of *cad* mRNA during fly development (Cho et al., 2005). Similarly, in *H. sapiens*, Hoxb4 translational repression relies on 4EHP (Villaescusa et al., 2009), and others have shown that 4EHP facilitates translational silencing by interacting with miRNA machinery (Chapat et al., 2017). Whether any given eIF4E isoform acts positively or negatively may be mRNA-dependent, or more likely due to the composition of the larger mRNP complex in which it resides.

Polyadenylation/deadenylation machinery and localization factors also rely on eIF4E:4EIP complexes to appropriately regulate their target mRNAs by repression/activation and localization (Cao and Richter, 2002; Wilhelm et al., 2003; Wong and Schedl, 2011; Romasko et al., 2013; Sengupta et al., 2013; Waghray et al., 2015; Davidson et al., 2016). Future studies will address what other proteins are necessary to push a repressed mRNP into an active translation complex in response to a developmental stimulus. A new understanding of mRNP dynamics will be important in providing a complete picture of how germ cells regulate gene expression by translational control. Technical advances that allow biochemical dissection of repressed vs. activated mRNPs isolated from germ cells in a variety of conditions (genetic mutants, developmental stage, intrinsic and extrinsic signaling, etc.) will aid in our understanding. One such RNA-proximity labeling technique, called APEX-Seq, can be used to determine not only spatial information about RNAs, but also their enrichment or depletion near RBPs of interest under varying conditions (Padron et al., 2016). APEX-Seq was used to elucidate how stress granules change in their RNA composition with different stressors, and the organization of 43S PIC complexes. Such advances will contribute greatly to our understanding of spatial and temporal post-transcriptional gene expression. Our comprehension of germ cell mRNA metabolism has made great strides in the past several decades, uncovering the roles of individual RBPs in mRNPs that inhibit translation. However, we must now begin to treat the whole mRNP as a discreet regulatory unit with both negative and positive roles, and probe deeper into what causes subsequent translational activation. This broader view will advance our understanding of post-transcriptional genetic switches and the unique cells that use them so extensively.

## Author Contributions

HH wrote and revised the manuscript and created the figures. BK assisted in writing and revising the manuscript.

## Conflict of Interest

The authors declare that the research was conducted in the absence of any commercial or financial relationships that could be construed as a potential conflict of interest.

## References

[B1] Amiri A. Keiper B. D. Kawasaki I. Fan Y. Kohara Y. Rhoads R. E. (2001). An isoform of eIF4E is a component of germ granules and is required for spermatogenesis in *C. elegans*. *Development* 128 3899–3912. 11641215 10.1242/dev.128.20.3899 PMC2430591

[B2] Andralojc K. M. Campbell A. C. Kelly A. L. Terrey M. Tanner P. C. Gans I. M. (2017). ELLI-1, a novel germline protein, modulates RNAi activity and P-granule accumulation in *Caenorhabditis elegans*. *PLoS Genet.* 13:e1006611 10.1371/journal.pone.01006611 PMC5325599 28182654

[B3] Baker C. C. Fuller M. T. (2007). Translational control of meiotic cell cycle progression and spermatid differentiation in male germ cells by a novel eIF4G homolog. *Development* 134 2863–2869. 10.1242/dev.003764 17611220 PMC4620998

[B4] Barkoff A. Ballantyne S. Wickens M. (1998). Meiotic maturation in *Xenopus* requires polyadenylation of multiple mRNAs. *EMBO J.* 17 3168–3175. 10.1093/emboj/17.11.3168 9606198 PMC1170655

[B5] Barkoff A. F. Dickson K. S. Gray N. K. Wickens M. (2000). Translational control of cyclin B1 mRNA during meiotic maturation: coordinated repression and cytoplasmic polyadenylation. *Development* 220 97–109. 10.1006/dbio.2000.9613 10720434

[B6] Barnard D. C. Ryan K. Manley J. L. Richter J. D. (2004). Symplekin and xGLD-2 are required for CPEB-mediated cytoplasmic polyadenylation. *Cell* 119 641–651. 10.1016/j.cell.2004.10.029 15550246

[B7] Becker K. Bluhm A. Casas-Vila N. Dinges N. Dejung M. Sayols S. (2018). Quantifying post-transcriptional regulation in the development of *Drosophila melanogaster*. *Nat. Commun.* 9:4970 10.1038/s41467-018-07455-7459 PMC6255845 30478415

[B8] Beretta L. Gingras A. C. Svitkin Y. V. Hall M. N. Sonenberg N. (1996). Rapamycin blocks the phosphorylation of 4E-BP1 and inhibits cap-dependent initiation of translation. *EMBO J.* 15 658–664. 10.1002/j.1460-2075.1996.tb00398.x 8599949 PMC449984

[B9] Bergsten S. E. Gavis E. R. (1999). Role for mRNA localization in translational activation but not spatial restriction of nanos RNA. *Development* 126 659–669. 9895314 10.1242/dev.126.4.659

[B10] Berthet C. Morera A. M. Asensio M. J. Chauvin M. A. Morel A. P. Dijoud F. (2004). CCR4-associated factor CAF1 is an essential factor for spermatogenesis. *Mol. Cell. Biol.* 24 5808–5820. 10.1128/MCB.24.13.5808-5820.2004 15199137 PMC480892

[B11] Bhandari D. Raisch T. Weichenrieder O. Jonas S. Izaurralde E. (2014). Structural basis for the Nanos-mediated recruitment of the CCR4-NOT complex and translational repression. *Genes. Dev.* 28 888–901. 10.1101/gad.237289.113 24736845 PMC4003280

[B12] Brangwynne C. P. Eckmann C. R. Courson D. S. Rybarska A. Hoege C. Gharakhani J. (2009). Germline P granules are liquid droplets that localize by controlled dissolution/condensation. *Science* 324 1729–1732. 10.1126/science.1172046 19460965

[B13] Busada J. T. Chappell V. A. Niedenberger B. A. Kaye E. P. Keiper B. D. Hogarth C. A. (2015). Retinoic acid regulates Kit translation during spermatogonial differentiation in the mouse. *Dev. Biol.* 397 140–149. 10.1016/j.ydbio.2014.10.020 25446031 PMC4268412

[B14] Campbell A. C. Updike D. L. (2015). CSR-1 and P granules suppress sperm-specific transcription in the *C. elegans* germline. *Development* 142 1745–1755. 10.1242/dev.121434 25968310 PMC4440928

[B15] Cao Q. Richter J. D. (2002). Dissolution of the maskin-eIF4E complex by cytoplasmic polyadenylation and poly(A)-binding protein controls cyclin B1 mRNA translation and oocyte maturation. *EMBO J.* 21 3852–3862. 10.1093/emboj/cdf353 12110596 PMC126103

[B16] Carthew R. W. Sontheimer E. J. (2009). Origins and mechanisms of miRNAs and siRNAs. *Cell* 136 642–655. 10.1016/j.cell.2009.01.035 19239886 PMC2675692

[B17] Casanal A. Kumar A. Hill C. H. Easter A. D. Emsley P. Degliesposti G. (2017). Architecture of eukaryotic mRNA 3’-end processing machinery. *Science* 358 1056–1059. 10.1126/science.aao6535 29074584 PMC5788269

[B18] Chapat C. Jafarnejad S. M. Matta-Camacho E. Hesketh G. G. Gelbart I. A. Attig J. (2017). Cap-binding protein 4EHP effects translation silencing by microRNAs. *Proc. Natl. Acad. Sci. U.S.A.* 114 5425–5430. 10.1073/pnas.1701488114 28487484 PMC5448183

[B19] Cho P. F. Poulin F. Cho-Park Y. A. Cho-Park I. B. Chicoine J. D. Lasko P. (2005). A new paradigm for translational control: inhibition via 5’-3’ mRNA tethering by Bicoid and the eIF4E cognate 4EHP. *Cell* 121 411–423. 10.1016/j.cell.2005.02.024 15882623

[B20] Clouse K. N. Ferguson S. B. Schupbach T. (2008). Squid, Cup, and PABP55B function together to regulate gurken translation in *Drosophila*. *Dev. Biol.* 313 713–724. 10.1016/j.ydbio.2007.11.008 18082158 PMC2276622

[B21] Collier B. Gorgoni B. Loveridge C. Cooke H. J. Gray N. K. (2005). The DAZL family proteins are PABP-binding proteins that regulate translation in germ cells. *EMBO J.* 24 2656–2666. 10.1038/sj.emboj.7600738 16001084 PMC1176464

[B22] Contreras V. Richardson M. A. Hao E. Keiper B. D. (2008). Depletion of the cap-associated isoform of translation factor eIF4G induces germline apoptosis in *C. elegans*. *Cell Death Differ.* 15 1232–1242. 10.1038/cdd.2008.46 18451872

[B23] Copeland P. R. Wormington M. (2001). The mechanism and regulation of deadenylation: identification and characterization of *Xenopus* PARN. *RNA* 7 875–886. 10.1017/s1355838201010020 11424938 PMC1370141

[B24] Cordeiro Rodrigues R. J. de Jesus Domingues A. M. Hellmann S. Dietz S. de Albuquerque B. F. M. Renz C. (2019). PETISCO is a novel protein complex required for 21U RNA biogenesis and embryonic viability. *Genes Dev.* 33 857–870. 10.1101/gad.322446.118 31147388 PMC6601512

[B25] Crucs S. Chatterjee S. Gavis E. R. (2000). Overlapping but distinct RNA elements control repression and activation of nanos translation. *Mol. Cell.* 5 457–467. 10.1016/s1097-2765(00)80440-80442 10882131

[B26] Cui J. Sartain C. V. Pleiss J. A. Wolfner M. F. (2013). Cytoplasmic polyadenylation is a major mRNA regulator during oogenesis and egg activation in *Drosophila*. *Dev. Biol.* 383 121–131. 10.1016/j.ydbio.2013.08.013 23978535 PMC3821703

[B27] Davidson A. Parton R. M. Rabouille C. Weil T. T. Davis I. (2016). Localized translation of gurken/TGF-alpha mRNA during axis specification is controlled by access to Orb/CPEB on processing bodies. *Cell Rep.* 14 2451–2462. 10.1016/j.celrep.2016.02.038 26947065 PMC4823467

[B28] Ding X. C. Grosshans H. (2009). Repression of *C. elegans* microRNA targets at the initiation level of translation requires GW182 proteins. *EMBO J.* 28 213–222. 10.1038/emboj.2008.275 19131968 PMC2637332

[B29] Dinkova T. D. Keiper B. D. Korneeva N. L. Aamodt E. J. Rhoads R. E. (2005). Translation of a small subset of *Caenorhabditis elegans* mRNAs is dependent on a specific eukaryotic translation initiation factor 4E isoform. *Mol. Cell. Biol.* 25 100–113. 10.1128/mcb.25.1.100-113.2005 15601834 PMC538781

[B30] Dufourt J. Bontonou G. Chartier A. Jahan C. Meunier A. C. Pierson S. (2017). piRNAs and aubergine cooperate with Wispy poly(A) polymerase to stabilize mRNAs in the germ plasm. *Nat. Commun.* 8:1305 10.1038/s41467-017-01431-1435 PMC5670238 29101389

[B31] Evsikov A. V. Graber J. H. Brockman J. M. Hampl A. Holbrook A. E. Singh P. (2006). Cracking the egg: molecular dynamics and evolutionary aspects of the transition from the fully grown oocyte to embryo. *Genes Dev.* 20 2713–2727. 10.1101/gad.1471006 17015433 PMC1578697

[B32] Feoktistova K. Tuvshintogs E. Do A. Fraser C. S. (2013). Human eIF4E promotes mRNA restructuring by stimulating eIF4A helicase activity. *Proc. Natl. Acad. Sci. U.S.A.* 110 13339–13344. 10.1073/pnas.1303781110 23901100 PMC3746923

[B33] Flemr M. Ma J. Schultz R. M. Svoboda P. (2010). P-body loss is concomitant with formation of a messenger RNA storage domain in mouse oocytes. *Biol. Reprod.* 82 1008–1017. 10.1095/biolreprod.109.082057 20075394 PMC2857638

[B34] Franklin-Dumont T. M. Chatterjee C. Wasserman S. A. Dinardo S. (2007). A novel eIF4G homolog, Off-schedule, couples translational control to meiosis and differentiation in *Drosophila spermatocytes*. *Development* 134 2851–2861. 10.1242/dev.003517 17611222

[B35] Freimer J. W. Hu T. J. Blelloch R. (2018). Decoupling the impact of microRNAs on translational repression versus RNA degradation in embryonic stem cells. *eLife* 7:38014. 10.7554/eLife.38014 30044225 PMC6086665

[B36] Friday A. J. Henderson M. A. Morrison J. K. Hoffman J. L. Keiper B. D. (2015). Spatial and temporal translational control of germ cell mRNAs mediated by the eIF4E isoform IFE-1. *J. Cell Sci.* 128 4487–4498. 10.1242/jcs.172684 26542024

[B37] Friday A. J. Keiper B. D. (2015). Positive mRNA translational control in germ cells by initiation factor selectivity. *Biomed. Res. Intern.* 2015:e327963. 10.1155/2015/327963 PMC4556832 26357652

[B38] Friend K. Campbell Z. T. Cooke A. Kroll-Conner P. Wickens M. P. Kimble J. (2012). A conserved PUF-Ago-eEF1A complex attenuates translation elongation. *Nat. Struct. Mol. Biol.* 19 176–183. 10.1038/nsmb.2214 22231398 PMC3293257

[B39] Gallo C. M. Munro E. Rasoloson D. Merritt C. Seydoux G. (2008). Processing bodies and germ granules are distinct RNA granules that interact in *C. elegans* embryos. *Dev. Biol.* 323 76–87. 10.1016/j.ydbio.2008.07.008 18692039

[B40] Gao M. Arkov A. L. (2013). Next generation organelles: structure and role of germ granules in the germline. *Mol. Reprod. Dev.* 80 610–623. 10.1002/mrd.22115 23011946 PMC3584238

[B41] Gingras A.-C. Raught B. Sonenberg N. (1999). eIF4 initiation factors: effectors of mRNA recruitment to ribosomes and regulators of translation. *Annu. Rev. Biochem.* 68 913–963. 10.1146/annurev.biochem.68.1.913 10872469

[B42] Haghighat A. Mader S. Pause A. Sonenberg N. (1995). Repression of cap-dependent translation by 4E-binding protein 1: competition with p220 for binding to eukaryotic initiation factor-4E. *EMBO J.* 14 5701–5709. 10.1002/j.1460-2075.1995.tb00257.x 8521827 PMC394685

[B43] Hake L. E. Richter J. D. (1994). CPEB is a specificity factor that mediates cytoplasmic polyadenylation during *Xenopus oocyte* maturation. *Cell* 79 617–627. 10.1016/0092-8674(94)90547-90549 7954828

[B44] Henderson M. A. Cronland E. Dunkelbarger S. Contreras V. Strome S. Keiper B. D. (2009). A germ line-specific isoform of eIF4E (IFE-1) is required for efficient translation of stored mRNAs and maturation of both oocytes and sperm. *J. Cell Sci.* 122 1529–1539. 10.1242/jcs.046771 19383718 PMC2680099

[B45] Hentze M. W. Castello A. Schwarzl T. Preiss T. (2018). A brave new world of RNA-binding proteins. *Nat. Rev. Mol. Cell Biol.* 19 327–341. 10.1038/nrm.2017.130 29339797

[B46] Hernandez G. Altmann M. Sierra J. M. Urlaub H. del Corral R. D. Schwartz P. (2005). Functional analysis of seven genes encoding eight translation initiation factor 4E (eIF4E) isoforms in *Drosophila*. *Mech. Dev.* 122 529–543. 10.1016/j.mod.2004.11.011 15804566

[B47] Hernandez G. Miron M. Han H. Liu N. Magescas J. Tettweiler G. (2013). Mextli is a novel eukaryotic translation initiation factor 4E-binding protein that promotes translation in *Drosophila melanogaster*. *Mol. Cell. Biol.* 33 2854–2864. 10.1128/mcb.01354-12 23716590 PMC3719689

[B48] Hershey J. W. Sonenberg N. Mathews M. B. (2012). Principles of translational control: an overview. *Cold Spring Harb. Perspect. Biol.* 4:a011528. 10.1101/cshperspect.a011528 23209153 PMC3504442

[B49] Hu J. Sun F. Handel M. A. (2018). Nuclear localization of EIF4G3 suggests a role for the XY body in translational regulation during spermatogenesis in mice. *Biol. Reprod.* 98 102–114. 10.1093/biolre/iox150 29161344 PMC5819848

[B50] Huang Y. Carmichael G. G. (1996). Role of polyadenylation in nucleocytoplasmic transport of mRNA. *Mol. Cell. Biol.* 16 1534–1542. 10.1128/mcb.16.4.1534 8657127 PMC231138

[B51] Huggins H. P. Subash J. S. Stoffel H. Henderson M. A. Hoffman J. L. Buckner D. S. (2020). Distinct roles of two eIF4E isoforms in the germline of *Caenorhabditis elegans*. *J. Cell. Sci.* 133:jcs237990. 10.1242/jcs.237990 32079657 PMC7132772

[B52] Igreja C. Izaurralde E. (2011). CUP promotes deadenylation and inhibits decapping of mRNA targets. *Genes Dev.* 25 1955–1967. 10.1101/gad.17136311 21937713 PMC3185967

[B53] Igreja C. Peter D. Weiler C. Izaurralde E. (2014). 4E-BPs require non-canonical 4E-binding motifs and a lateral surface of eIF4E to repress translation. *Nat. Commun.* 5:4790. 10.1038/ncomms5790 25179781 PMC4164784

[B54] Iwakawa H. O. Tomari Y. (2015). The functions of MicroRNAs: mRNA decay and translational repression. *Trends Cell Biol.* 25 651–665. 10.1016/j.tcb.2015.07.011 26437588

[B55] Jankowska-Anyszka M. Lamphear B. J. Aamodt E. J. Harrington T. Darzynkiewicz E. Stolarski R. (1998). Multiple isoforms of eukaryotic protein synthesis initiation factor 4E in *Caenorhabditis elegans* can distinguish between, mono- and trimethylated mRNA cap structures. *J. Biol. Chem.* 273 10538–10542. 10.1074/jbc.273.17.10538 9553113

[B56] Kahvejian A. Roy G. Sonenberg N. (2001). The mRNA closed-loop model: the function of PABP and PABP-interacting proteins in mRNA translation. *Cold. Spring Harb. Symp. Quant. Biol.* 66 293–300. 10.1101/sqb.2001.66.293 12762031

[B57] Kawasaki I. Jeong M. H. Shim Y. H. (2011). Regulation of sperm-specific proteins by IFE-1, a germline-specific homolog of eIF4E, in *C. elegans*. *Mol. Cells* 31 191–197. 10.1007/s10059-011-0021-y 21191815 PMC3932688

[B58] Kawasaki I. Shim Y. H. Kirchner J. Kaminker J. Wood W. B. Strome S. (1998). PGL-1, a predicted RNA-binding component of germ granules, is essential for fertility in *C. elegans*. *Cell* 94 635–645. 10.1016/s0092-8674(00)81605-0 9741628

[B59] Keiper B. (2019). Cap-independent mRNA translation in germ cells. *Int. J. Mol. Sci.* 20:173. 10.3390/ijms20010173 30621249 PMC6337596

[B60] Keiper B. D. Lamphear B. J. Deshpande A. M. Jankowska-Anyszka M. Aamodt E. J. Blumenthal T. (2000). Functional characterization of five eIF4E isoforms in *Caenorhabditis elegans*. *J. Biol. Chem.* 275 10590–10596. 10.1074/jbc.275.14.10590 10744754

[B61] Kevil C. G. De Benedetti A. Payne D. K. Coe L. L. Laroux F. S. Alexander J. S. (1996). Translational regulation of vascular permeability factor by eukaryotic initiation factor 4E: implications for tumor angiogenesis. *Int. J. Cancer* 65 785–790. 10.1002/(sici)1097-0215(19960315)65:6<785::aid-ijc14>3.0.co;2-3 8631593

[B62] Kim J. H. Richter J. D. (2006). Opposing polymerase-deadenylase activities regulate cytoplasmic polyadenylation. *Mol. Cell.* 24 173–183. 10.1016/j.molcel.2006.08.016 17052452

[B63] Kim K. W. Wilson T. L. Kimble J. (2010). GLD-2/RNP-8 cytoplasmic poly(A) polymerase is a broad-spectrum regulator of the oogenesis program. *Proc. Natl. Acad. Sci. U.S.A.* 107 17445–17450. 10.1073/pnas.1012611107 20855596 PMC2951458

[B64] Kim-Ha J. Smith J. L. Macdonald P. M. (1991). oskar mRNA is localized to the posterior pole of the *Drosophila oocyte*. *Cell* 66 23–35. 10.1016/0092-8674(91)90136-m 2070416

[B65] Komar A. A. Hatzoglou M. (2011). Cellular IRES-mediated translation: the war of ITAFs in pathophysiological states. *Cell* 10 229–240. 10.4161/cc.10.2.14472 21220943 PMC3048795

[B66] Kotaja N. Bhattacharyya S. N. Jaskiewicz L. Kimmins S. Parvinen M. Filipowicz W. (2006). The chromatoid body of male germ cells: similarity with processing bodies and presence of dicer and microRNA pathway components. *Proc. Natl. Acad. Sci. U.S.A.* 103 2647–2652. 10.1073/pnas.0509333103 16477042 PMC1413789

[B67] Lahr R. M. Fonseca B. D. Ciotti G. E. Al-Ashtal H. A. Jia J. J. Niklaus M. R. (2017). La-related protein 1 (LARP1) binds the mRNA cap, blocking eIF4F assembly on TOP mRNAs. *eLife* 6:24146. 10.7554/eLife.24146 28379136 PMC5419741

[B68] Lasko P. (2012). mRNA localization and translational control in *Drosophila oogenesis*. *Cold Spring Harb. Perspect. Biol.* 4:cshersect.a012294. 10.1101/cshperspect.a012294 22865893 PMC3475173

[B69] Lebedeva L. A. Yakovlev K. V. Kozlov E. N. Schedl P. Deshpande G. Shidlovskii Y. V. (2018). Transcriptional quiescence in primordial germ cells. *Crit. Rev. Biochem. Mol. Biol.* 53 579–595. 10.1080/10409238.2018.1506733 30280955 PMC8729227

[B70] Lee M. H. Mamillapalli S. S. Keiper B. D. Cha D. S. (2015). A systematic mRNA control mechanism for germline stem cell homeostasis and cell fate specification. *BMB Rep.* 2015:3259. 10.5483/BMBRep.2016.49.2.135 PMC4915122 26303971

[B71] MacDougall N. Clark A. MacDougall E. Davis I. (2003). *Drosophila gurken* (TGFalpha) mRNA localizes as particles that move within the oocyte in two dynein-dependent steps. *Dev. Cell* 4 307–319. 10.1016/s1534-5807(03)00058-3 12636913

[B72] Mader S. Lee H. Pause A. Sonenberg N. (1995). The translation initiation factor eIF-4E binds to a common motif shared by the translation factor eIF-4 gamma and the translational repressors 4E-binding proteins. *Mol. Cell. Biol.* 15 4990–4997. 10.1128/mcb.15.9.4990 7651417 PMC230746

[B73] Magnuson B. Ekim B. Fingar D. C. (2012). Regulation and function of ribosomal protein S6 kinase (S6K) within mTOR signalling networks. *Biochem. J.* 441 1–21. 10.1042/BJ20110892 22168436

[B74] Mahowald A. P. Hennen S. (1971). Ultrastructure of the “germ plasm” in eggs and embryos of *Rana pipiens*. *Dev. Biol.* 24 37–53. 10.1016/0012-1606(71)90045-90045 4107682

[B75] Mancera-Martinez E. Brito Querido J. Valasek L. S. Simonetti A. Hashem Y. (2017). ABCE1: A special factor that orchestrates translation at the crossroad between recycling and initiation. *RNA Biol.* 14 1279–1285. 10.1080/15476286.2016.1269993 28498001 PMC5711452

[B76] Marnik E. A. Fuqua J. H. Sharp C. S. Rochester J. D. Xu E. L. Holbrook S. E. (2019). Germline maintenance through the multifaceted activities of GLH/Vasa in *Caenorhabditis elegans* P granules. *Genetics* 213 923–939. 10.1534/genetics.119.302670 31506335 PMC6827368

[B77] Mayberry L. K. Allen M. L. Dennis M. D. Browning K. S. (2009). Evidence for variation in the optimal translation initiation complex: plant eIF4B, eIF4F, and eIF(iso)4F differentially promote translation of mRNAs. *Plant Physiol.* 150 1844–1854. 10.1104/pp.109.138438 19493973 PMC2719132

[B78] Mendez R. Hake L. E. Andresson T. Littlepage L. E. Ruderman J. V. Richter J. D. (2000). Phosphorylation of CPE binding factor by Eg2 regulates translation of c-mos mRNA. *Nature* 404 302–307. 10.1038/35005126 10749216

[B79] Mendez R. Richter J. D. (2001). Translational control by CPEB: a means to the end. *Nat. Rev. Mol. Cell Biol.* 2 521–529. 10.1038/35080081 11433366

[B80] Minshall N. Reiter M. H. Weil D. Standart N. (2007). CPEB interacts with an ovary-specific eIF4E and 4E-T in early *Xenopus oocytes*. *J. Biol. Chem.* 282 37389–37401. 10.1074/jbc.m704629200 17942399

[B81] Mitchell S. F. Parker R. (2014). Principles and properties of eukaryotic mRNPs. *Mol. Cell.* 54 547–558. 10.1016/j.molcel.2014.04.033 24856220

[B82] Mohr S. E. Dillon S. T. Boswell R. E. (2001). The RNA-binding protein tsunagi interacts with mago nashi to establish polarity and localize oskar mRNA during *Drosophila oogenesis*. *Genes Dev.* 15 2886–2899. 10.1101/gad.927001 11691839 PMC312802

[B83] Morrison J. K. Friday A. J. Henderson M. A. Hao E. Keiper B. D. (2014). Induction of cap-independent BiP (hsp-3) and Bcl-2 (ced-9) translation in response to eIF4G (IFG-1) depletion in *C. elegans*. *Translation* 2:e28935. 10.4161/trla.28935 26779406 PMC4705828

[B84] Nakamura A. Sato K. Hanyu-Nakamura K. (2004). *Drosophila* cup is an eIF4E binding protein that associates with Bruno and regulates oskar mRNA translation in oogenesis. *Dev. Cell* 6 69–78. 10.1016/s1534-5807(03)00400-3 14723848

[B85] Nelson M. R. Leidal A. M. Smibert C. A. (2004). *Drosophila* Cup is an eIF4E-binding protein that functions in Smaug-mediated translational repression. *EMBO J.* 23 150–159. 10.1038/sj.emboj.7600026 14685270 PMC1271664

[B86] Nijjar S. Woodland H. R. (2013). Localisation of RNAs into the germ plasm of vitellogenic *Xenopus oocytes*. *PLoS One* 8:e61847. 10.1371/journal.pone.0061847 23626739 PMC3633952

[B87] Nousch M. Eckmann C. R. (2013). Translational control in the *Caenorhabditis elegans* germ line. *Adv. Exp. Med. Biol.* 757 205–247. 10.1007/978-1-4614-4015-4_8 22872479

[B88] Nousch M. Techritz N. Hampel D. Millonigg S. Eckmann C. R. (2013). The Ccr4-Not deadenylase complex constitutes the main poly(A) removal activity in *C. elegans*. *J. Cell Sci.* 126 4274–4285. 10.1242/jcs.132936 23843623

[B89] Okamura K. Ishizuka A. Siomi H. Siomi M. C. (2004). Distinct roles for Argonaute proteins in small RNA-directed RNA cleavage pathways. *Genes Dev.* 18 1655–1666. 10.1101/gad.1210204 15231716 PMC478188

[B90] Ottone C. Gigliotti S. Giangrande A. Graziani F. Verrotti di Pianella A. (2012). The translational repressor cup is required for germ cell development in *Drosophila*. *J. Cell Sci.* 125 3114–3123. 10.1242/jcs.095208 22454519

[B91] Ouyang J. P. T. Folkmann A. Bernard L. Lee C. Y. Seroussi U. Charlesworth A. G. (2019). P granules protect RNA interference genes from silencing by piRNAs. *Dev. Cell* 50 716–728. 10.1016/j.devcel.2019.07.026 31402283 PMC6764750

[B92] Padron A. Iwasaki S. Ingolia N. T. (2016). Proximity RNA labeling by APEX-Seq reveals the organization of translation initiation complexes and repressive RNA granules. *Mol. Cell.* 75 875–887. 10.1016/j.molcel.2019.07.030 PMC6834362 31442426

[B93] Paillard L. Maniey D. Lachaume P. Legagneux V. Osborne H. B. (2000). Identification of a C-rich element as a novel cytoplasmic polyadenylation element in *Xenopus embryos*. *Mech. Dev.* 93 117–125. 10.1016/s0925-4773(00)00279-273 10781945

[B94] Parma D. H. Bennett P. E. J. Boswell R. E. (2007). Mago Nashi and Tsunagi/Y14, respectively, regulate *Drosophila germline* stem cell differentiation and oocyte specification. *Dev. Biol.* 308 507–519. 10.1016/j.ydbio.2007.06.007 17628520 PMC3010412

[B95] Pique M. Lopez J. M. Foissac S. Guigo R. Mendez R. (2008). A combinatorial code for CPE-mediated translational control. *Cell* 132 434–448. 10.1016/j.cell.2007.12.038 18267074

[B96] Prasad A. Porter D. F. Kroll-Conner P. L. Mohanty I. Ryan A. R. Crittenden S. L. (2016). The PUF binding landscape in metazoan germ cells. *RNA* 22 1026–1043. 10.1261/rna.055871.116 27165521 PMC4911911

[B97] Puoti A. Pugnale P. Belfiore M. Schlappi A. C. Saudan Z. (2001). RNA and sex determination in *Caenorhabditis elegans*. Post-transcriptional regulation of the sex-determining *tra-*2 and fem-3 mRNAs in the *Caenorhabditis elegans* hermaphrodite. *EMBO Rep.* 2 899–904. 10.1093/embo-reports/kve209 11600454 PMC1084087

[B98] Pushpa K. Kumar G. A. Subramaniam K. (2017). Translational control of germ cell decisions. *Results Probl. Cell Differ.* 59 175–200. 10.1007/978-3-319-44820-6_6 28247049 PMC5985952

[B99] Raesch F. Weber R. Izaurralde E. Igreja C. (2020). 4E-T-bound mRNAs are stored in a silenced and deadenylated form. *Genes Dev.* 34 847–860. 10.1101/gad.336073.119 32354837 PMC7263148

[B100] Reed K. J. Svendsen J. M. Brown K. C. Montgomery B. E. Marks T. N. Vijayasarathy T. (2020). Widespread roles for piRNAs and WAGO-class siRNAs in shaping the germline transcriptome of *Caenorhabditis elegans*. *Nucleic Acids Res.* 48 1811–1827. 10.1093/nar/gkz1178 31872227 PMC7038979

[B101] Rhoads R. E. Joshi-Barve S. Rinker-Schaeffer C. (1993). Mechanism of action and regulation of protein synthesis initiation factor 4E: effects on mRNA discrimination, cellular growth rate, and oncogenesis. *Progr. Nucl. Acid Res. Mol. Biol.* 46 183–219. 10.1016/s0079-6603(08)61022-3 8234784

[B102] Richter J. D. (1999). Cytoplasmic polyadenylation in development and beyond. *Microbiol. Mol. Biol. Rev.* 63 446–456. 10.1128/mmbr.63.2.446-456.1999 10357857 PMC98972

[B103] Rodriguez C. M. Freire M. A. Camilleri C. Robaglia C. (1998). The *Arabidopsis thaliana* cDNAs coding for eIF4E and eIF(iso)4E are not functionally equivalent for yeast complementation and are differentially expressed during plant development. *Plant J.* 13 465–473. 10.1046/j.1365-313x.1998.00047.x 9680993

[B104] Romasko E. J. Amarnath D. Midic U. Latham K. E. (2013). Association of maternal mRNA and phosphorylated EIF4EBP1 variants with the spindle in mouse oocytes: localized translational control supporting female meiosis in mammals. *Genetics* 195 349–358. 10.1534/genetics.113.154005 23852387 PMC3781964

[B105] Rosenwald I. B. Kaspar R. Rousseau D. Gehrke L. Leboulch P. Chen J. J. (1995). Eukaryotic translation initiation factor 4E regulates expression of cyclin D1 at transcriptional and post-transcriptional levels. *J. Biol. Chem.* 270 21176–21180. 10.1074/jbc.270.36.21176 7673150

[B106] Rouget C. Papin C. Boureux A. Meunier A. C. Franco B. Robine N. (2010). Maternal mRNA deadenylation and decay by the piRNA pathway in the early *Drosophila embryo*. *Nature* 467 1128–1132. 10.1038/nature09465 20953170 PMC4505748

[B107] Rozen F. Edery I. Meerovitch K. Dever T. E. Merrick W. C. Sonenberg N. (1990). Bidirectional RNA helicase activity of eucaryotic translation initiation factors 4A and 4F. *Mol. Cell. Biol.* 10 1134–1144. 10.1128/mcb.10.3.1134 2304461 PMC360981

[B108] Salaun P. Pyronnet S. Morales J. Mulner-Lorillon O. Belle R. Sonenberg N. (2003). eIF4E/4E-BP dissociation and 4E-BP degradation in the first mitotic division of the sea urchin embryo. *Dev. Biol.* 255 428–439. 10.1016/s0012-1606(02)00099-94 12648502

[B109] Saxe J. P. Lin H. (2011). Small noncoding RNAs in the germline. *Cold Spring Harb. Perspect. Biol.* 3:a002717. 10.1101/cshperspect.a002717 21669983 PMC3181032

[B110] Schier A. F. (2007). The maternal-zygotic transition: death and birth of RNAs. *Science* 316 406–407. 10.1126/science.1140693 17446392

[B111] Schisa J. A. (2019). Germ cell responses to stress: the role of RNP granules. *Front. Cell Dev. Biol.* 7:220. 10.3389/fcell.2019.00220 31632971 PMC6780003

[B112] Schisa J. A. Pitt J. N. Priess J. R. (2001). Analysis of RNA associated with P granules in germ cells of *C. elegans* adults. *Development* 128 1287–1298. 11262230 10.1242/dev.128.8.1287

[B113] Sen N. D. Zhou F. Harris M. S. Ingolia N. T. Hinnebusch A. G. (2016). eIF4B stimulates translation of long mRNAs with structured 5’ UTRs and low closed-loop potential but weak dependence on eIF4G. *Proc. Natl. Acad. Sci. U.S.A.* 113 10464–10472. 10.1073/pnas.1612398113 27601676 PMC5035867

[B114] Sengupta M. S. Boag P. R. (2012). Germ granules and the control of mRNA translation. *IUBMB Life* 64 586–594. 10.1002/iub.1039 22639345

[B115] Sengupta M. S. Low W. Y. Patterson J. R. Kim H. M. Traven A. Beilharz T. H. (2013). ifet-1 is a broad-scale translational repressor required for normal P granule formation in *C. elegans*. *J. Cell Sci.* 126 850–859. 10.1242/jcs.119834 23264733 PMC3619813

[B116] Sheets M. D. Wu M. Wickens M. (1995). Polyadenylation of c-mos mRNA as a control point in *Xenopus meiotic* maturation. *Nature* 374 511–516. 10.1038/374511a0 7700377

[B117] Sheth U. Pitt J. Dennis S. Priess J. R. (2010). Perinuclear P granules are the principal sites of mRNA export in adult *C. elegans* germ cells. *Development* 137 1305–1314. 10.1242/dev.044255 20223759 PMC2847466

[B118] Simon R. Tassan J. P. Richter J. D. (1992). Translational control by poly(A) elongation during *Xenopus* development: differential repression and enhancement by a novel cytoplasmic polyadenylation element. *Genes Dev.* 6 2580–2591. 10.1101/gad.6.12b.2580 1285126

[B119] Slevin M. K. Gourronc F. Hartley R. S. (2007). ElrA binding to the 3’UTR of cyclin E1 mRNA requires polyadenylation elements. *Nucleic Acids Res.* 35 2167–2176. 10.1093/nar/gkm084 17355986 PMC1874641

[B120] Sonenberg N. Gingras A.-C. (1998). The mRNA 5’ cap-binding protein eIF4E and control of cell growth. *Curr. Opin. Cell Biol.* 10 268–275. 10.1016/s0955-0674(98)80150-6 9561852

[B121] Song A. Labella S. Korneeva N. L. Keiper B. D. Aamodt E. J. Zetka M. (2010). A *C. elegans eIF*4E-family member upregulates translation at elevated temperatures of mRNAs encoding MSH-5 and other meiotic crossover proteins. *J. Cell Sci.* 123 2228–2237. 10.1242/jcs.063107 20530576 PMC2886744

[B122] Spike C. A. Coetzee D. Nishi Y. Guven-Ozkan T. Oldenbroek M. Yamamoto I. (2014). Translational control of the oogenic program by components of OMA ribonucleoprotein particles in *Caenorhabditis elegans*. *Genetics* 198 1513–1533. 10.1534/genetics.114.168823 25261697 PMC4256769

[B123] Standart N. Minshall N. (2008). Translational control in early development: CPEB, P-bodies and germinal granules. *Biochem. Soc. Trans.* 36 671–676. 10.1042/BST0360671 18631138

[B124] Stebbins-Boaz B. Cao Q. de Moor C. H. Mendez R. Richter J. D. (1999). Maskin is a CPEB-associated factor that transiently interacts with elF-4E. *Mol. Cell* 4 1017–1027. 10.1016/s1097-2765(00)80230-0 10635326

[B125] Stebbins-Boaz B. Hake L. E. Richter J. D. (1996). CPEB controls the cytoplasmic polyadenylation of cyclin, Cdk2 and c-mos mRNAs and is necessary for oocyte maturation in *Xenopus*. *EMBO J.* 15 2582–2592. 10.1002/j.1460-2075.1996.tb00616.x 8665866 PMC450191

[B126] Suter B. (2018). RNA localization and transport. *Biochim. Biophys. Acta Gene Regul. Mech.* 1861 938–951. 10.1016/j.bbagrm.2018.08.004 30496039

[B127] Temme C. Zaessinger S. Meyer S. Simonelig M. Wahle E. (2004). A complex containing the CCR4 and CAF1 proteins is involved in mRNA deadenylation in *Drosophila*. *EMBO J.* 23 2862–2871. 10.1038/sj.emboj.7600273 15215893 PMC514940

[B128] Tettweiler G. Kowanda M. Lasko P. Sonenberg N. Hernández G. (2012). The distribution of eIF4E-family members across insecta. *Comparat. Funct. Genom.* 2012 1–15. 10.1155/2012/960420 22745595 PMC3382400

[B129] Thomson T. Liu N. Arkov A. Lehmann R. Lasko P. (2008). Isolation of new polar granule components in *Drosophila* reveals P body and ER associated proteins. *Mech. Dev.* 125 865–873. 10.1016/j.mod.2008.06.005 18590813 PMC2570953

[B130] Updike D. L. Knutson A. K. Egelhofer T. A. Campbell A. C. Strome S. (2014). Germ-granule components prevent somatic development in the *C. elegans germline*. *Curr. Biol.* 24 970–975. 10.1016/j.cub.2014.03.015 24746798 PMC4036631

[B131] Vazquez-Pianzola P. Urlaub H. Suter B. (2011). Pabp binds to the osk 3’UTR and specifically contributes to osk mRNA stability and oocyte accumulation. *Dev. Biol.* 357 404–418. 10.1016/j.ydbio.2011.07.009 21782810

[B132] Vi S. L. Trost G. Lange P. Czesnick H. Rao N. Lieber D. (2013). Target specificity among canonical nuclear poly(A) polymerases in plants modulates organ growth and pathogen response. *Proc. Natl. Acad. Sci. U.S.A.* 110 13994–13999. 10.1073/pnas.1303967110 23918356 PMC3752211

[B133] Villaescusa J. C. Buratti C. Penkov D. Mathiasen L. Planaguma J. Ferretti E. (2009). Cytoplasmic Prep1 interacts with 4EHP inhibiting Hoxb4 translation. *PLoS One* 4:e5213. 10.1371/journal.pone.0005213 19365557 PMC2664923

[B134] Voronina E. Paix A. Seydoux G. (2012). The P granule component PGL-1 promotes the localization and silencing activity of the PUF protein FBF-2 in germline stem cells. *Development* 139 3732–3740. 10.1242/dev.083980 22991439 PMC3445306

[B135] Voronina E. Seydoux G. Sassone-Corsi P. Nagamori I. (2011). RNA granules in germ cells. *Cold Spring Harb. Perspect. Biol.* 3:cshersect.a002774. 10.1101/cshperspect.a002774 21768607 PMC3225947

[B136] Waghray S. Williams C. Coon J. J. Wickens M. (2015). *Xenopus* CAF1 requires NOT1-mediated interaction with 4E-T to repress translation in vivo. *RNA* 21 1335–1345. 10.1261/rna.051565.115 26015597 PMC4478352

[B137] Wahle E. (1995). Poly(A) tail length control is caused by termination of processive synthesis. *J. Biol. Chem.* 270 2800–2808. 10.1074/jbc.270.6.2800 7852352

[B138] Wakiyama M. Imataka H. Sonenberg N. (2000). Interaction of eIF4G with poly(A)-binding protein stimulates translation and is critical for *Xenopus oocyte* maturation. *Curr. Biol.* 10 1147–1150. 10.1016/s0960-9822(00)00701-706 10996799

[B139] Wan G. Fields B. D. Spracklin G. Shukla A. Phillips C. M. Kennedy S. (2018). Spatiotemporal regulation of liquid-like condensates in epigenetic inheritance. *Nature* 557 679–683. 10.1038/s41586-018-0132-0 29769721 PMC6479227

[B140] Weick E. M. Miska E. A. (2014). piRNAs: from biogenesis to function. *Development* 141 3458–3471. 10.1242/dev.094037 25183868

[B141] Wells S. E. Hillner P. E. Vale R. D. Sachs A. B. (1998). Circularization of mRNA by eukaryotic translation initiation factors. *Mol. Cell* 2 135–140. 10.1016/s1097-2765(00)80122-7 9702200

[B142] Wilhelm J. E. Hilton M. Amos Q. Henzel W. J. (2003). Cup is an eIF4E binding protein required for both the translational repression of oskar and the recruitment of Barentsz. *J. Cell Biol.* 163 1197–1204. 10.1083/jcb.200309088 14691132 PMC2173729

[B143] Wong L. C. Schedl P. (2011). Cup blocks the precocious activation of the orb autoregulatory loop. *PLoS One* 6:e28261. 10.1371/journal.pone.0028261 22164257 PMC3229553

[B144] Wormington M. (1993). Poly(A) and translation: developmental control. *Curr. Opin. Cell Biol.* 5 950–954. 10.1016/0955-0674(93)90075-2 7907491

[B145] Wu Y. Han B. Gauvin T. J. Smith J. Singh A. Griffin E. E. (2019). Single-molecule dynamics of the P granule scaffold MEG-3 in the *Caenorhabditis elegans* zygote. *Mol. Biol. Cell* 30 333–345. 10.1091/mbc.E18-06-0402 30540524 PMC6589573

[B146] Yi H. Park J. Ha M. Lim J. Chang H. Kim V. N. (2018). PABP cooperates with the CCR4-NOT complex to promote mRNA deadenylation and block precocious decay. *Mol. Cell.* 70 1081–1088. 10.1016/j.molcel.2018.05.009 29932901

[B147] Yoffe Y. Zuberek J. Lerer A. Lewdorowicz M. Stepinski J. Altmann M. (2006). Binding specificities and potential roles of isoforms of eukaryotic initiation factor 4E in leishmania. *Eukaryot. Cell* 5 1969–1979. 10.1128/ec.00230-06 17041189 PMC1694823

[B148] Yu C. Ji S. Y. Sha Q. Q. Dang Y. Zhou J. J. Zhang Y. L. (2016). BTG4 is a meiotic cell cycle-coupled maternal-zygotic-transition licensing factor in oocytes. *Nat. Struct. Mol. Biol.* 23 387–394. 10.1038/nsmb.3204 27065194

[B149] Zaessinger S. Busseau I. Simonelig M. (2006). Oskar allows nanos mRNA translation in *Drosophila embryos* by preventing its deadenylation by Smaug/CCR4. *Development* 133 4573–4583. 10.1242/dev.02649 17050620

[B150] Zappavigna V. Piccioni F. Villaescusa J. C. Verrotti A. C. (2004). Cup is a nucleocytoplasmic shuttling protein that interacts with the eukaryotic translation initiation factor 4E to modulate *Drosophila* ovary development. *Proc. Natl. Acad. Sci. U.S.A.* 101 14800–14805. 10.1073/pnas.0406451101 15465908 PMC522052

